# Design and evaluation of a software for the objective and easy-to-read presentation of new drug properties to physicians

**DOI:** 10.1186/s12911-015-0158-2

**Published:** 2015-05-30

**Authors:** Maia Iordatii, Alain Venot, Catherine Duclos

**Affiliations:** INSERM, U1142, LIMICS, F-75006 Paris, France; Université Paris 13, Sorbonne Paris Cité, F-93000 Bobigny, France; Sorbonne Universités, Universités Paris, 06, F-75006 Paris, France

## Abstract

**Background:**

When new pharmaceutical products appear on the market, physicians need to know whether they are likely to be useful in their practices. Physicians currently obtain most of their information about the market release and properties of new drugs from pharmaceutical industry representatives. However, the official information contained in the summary of product characteristics (SPCs) and evaluation reports from health agencies, provide a more complete view of the potential value of new drugs, although they can be long and difficult to read. The main objective of this work was to design a prototype computer program to facilitate the objective appraisal of the potential value of a new pharmaceutical product by physicians. This prototype is based on the modeling of pharmaceutical innovations described in a previous paper.

**Methods:**

The interface was designed to allow physicians to develop a rapid understanding of the value of a new drug for their practices. We selected five new pharmaceutical products, to illustrate the function of this prototype. We considered only the texts supplied by national or international drug agencies at the time of market release. The perceived usability of the prototype was evaluated qualitatively, except for the System Usability Scale (SUS) score evaluation, by 10 physicians differing in age and medical background.

**Results:**

The display is based on the various axes of the conceptual model of pharmaceutical innovations. The user can select three levels of detail when consulting this information (highly synthetic, synthetic and detailed). Tables provide a comparison of the properties of the new pharmaceutical product with those of existing drugs, if available for the same indication, in terms of efficacy, safety and ease of use.

The interface was highly appreciated by evaluators, who found it easy to understand and suggested no other additions of important, internationally valid information. The mean System Usability Scale score for the 10 physicians was 82, corresponding to a “good” user interface.

**Conclusions:**

This work led us to propose the selection, grouping, and mode of presentation for various types of knowledge on pharmaceutical innovations in a way that was appreciated by evaluators. It provides physicians with readily accessible objective information about new drugs.

**Electronic supplementary material:**

The online version of this article (doi:10.1186/s12911-015-0158-2) contains supplementary material, which is available to authorized users.

## Background

When new pharmaceutical products are released onto the market, physicians need to determine whether they are likely to be useful in their own practices. Physicians currently obtain most of their information about the market release and properties of new drugs from pharmaceutical industry representatives [1]. The information provided by the pharmaceutical industry has been shown to have a strong influence on the prescription practices of general practitioners (GPs) [2,3]. This information generally gives a very positive image of the products concerned [4,5]. Unfortunately, various studies [6] have shown that the quality of prescription by GPs is negatively correlated with the frequency of visits from representatives of the pharmaceutical industry.

Physicians need to develop their own ideas about the value of new drugs. They may consider that a new drug is outside their specialty and therefore not of any interest for their patients. Alternatively, they may be curious about the potential value of a new drug of for their practice, but their decision to reject or accept it will be based on its efficacy and safety.

Scientific papers presenting the results of clinical trials and evaluation reports produced by health agencies are publicly available via the Internet and can help doctors to determine the efficacy of a new drug, but they are time-consuming to read.

Physicians can compare the safety aspects of new drugs with those of other drugs already used for the same indication, by analyzing the content of the summary of product characteristics (SPC) and drug monographs to check for contraindications, interactions, precautions, warnings and adverse reactions. However, it would be unrealistic to expect physicians to carry out such a task, which would be both difficult and time-consuming.

Various attempts have been made to increase the understanding and use of safety information about drugs.

Commercial editors of drug databases have developed systems for comparing the safety aspects of the SPCs of two drugs, by contrasting the contraindications or side effects of the drugs compared, for example. However, the information provided is still time-consuming to read and would benefit from synthetic, and possibly graphical approaches to decrease the cognitive burden of the comparison of textual elements of this kind.

An iconic language was developed in a previous study [7], for the presentation of medical concepts. This language has been applied to the presentation of drug contraindications and interactions and has been shown to make the reading of drug monographs more rapid [8].

Software has been created to improve medication safety in an emergency department, by enhancing the integration and presentation of safety information for drug treatments [9]. This decision support system alerts the user to potential drug interactions and contraindications. It is a four-step system, with the last step providing access to the most detailed information about the drug. It was developed to decrease significantly the time required for doctors to obtain documentation about drugs.

Drug fact boxes, a concept recently developed in the USA, provide valuable synthetic and comparative information about the efficacy and safety of drugs. These documents have been shown to be effective for patient information [10,11], but are not detailed enough for physicians. The comparative information is easy to read, but succinct. Several topics of importance to the physician [12] are not considered: the type of originality of the new drug (e.g. mechanism of action, pharmacotherapeutic group, galenic innovation) and its ease of use (e.g. existence of an antidote, need for laboratory tests during treatment).

A tool providing physicians with comparative drug information would be useful, if it provided both synthetic and detailed information about new drugs, comparing them with existing drugs for the same indication. Such a tool could be extended to cover all drugs, both new and older, but the development of such a tool might be difficult, time-consuming and costly, because there are many commercial products, evaluated at different times, some as long as 40 years ago, with different protocols and evaluation criteria.

Our main objective in this study was to design a prototype computer program to assist physicians in their evaluation of the potential value of new pharmaceutical products. This tool was designed to be used periodically by physicians for training, but not in front of patients.

This computer program was based on a previously described model [12], in which we identified the key items of information required for a fair appraisal of pharmaceutical innovations. We modeled this information, by grouping these items into three categories of information: (i) the medical context of use of the new pharmaceutical product, including the therapeutic arsenal for the same indication (the therapeutic arsenal is the entire set of drugs that may be prescribed for a given indication); (ii) the description of the pharmaceutical characteristics of the innovation in terms of its chemical, pharmacological and galenic properties; (iii) the expected impact of the new drug in terms of efficacy, safety and ease of use for the patient and the physician, as determined by comparison with other products for the same indication.

The interface was designed to enable the physician to develop a rapid understanding of the value of a new drug for his or her practice and should encourage physicians to go into greater detail and to analyze the available information for drugs identified as real innovations.

We will first present our methodological approach, which includes several levels of information synthesis, carried out as objectively as possible. We will then describe the design of the software and present the principal results for its qualitative evaluation.

## Methods

### Overall approach

In a previous study [12], we developed a model illustrating the basic features of a new pharmaceutical product. This model was constructed from the knowledge contained in the evaluation reports of the French National Authority for Health (HAS) and the SPCs of 40 drugs approved from 1 January 2008 to 1 January 2011. An initial set of innovation axes was selected by two authors. The model was then gradually developed through the reading of evaluation reports and of the SPCs of new drugs and of the drugs to which these new drugs were compared in the evaluation report for the items relating to drug impact, and the SPCs of all the drugs of the available therapeutic arsenal for items assessing novelty. We described the context of use of a new drug, its type of novelty, and elements influencing the impact (e.g. efficacy, safety, ease of use) of the new manufactured product with respect to a comparator. We follow the same separation of information in the development of the new drug presentation.

The interface must initially provide the physician with a first level of information granularity. An intermediate level should then provide more information, but should remain synthetic in nature, with elements that can be read rapidly. The third level of granularity should involve the display of more detailed information, including, in particular, excerpts of the evaluation report [13] and SPC. Recommendations have been made concerning the use of multiple views in information visualization, to illustrate different levels of abstraction [14].

We therefore created a first interface element, to provide a global description of the innovative features of any pharmaceutical product. We then decided to develop specific interfaces providing detailed information about each of the axes of innovation in the model presented in our previous study [12]. The interface is represented with stable elements, to avoid the need for learning.

### Choice of the type of graphical representation of information

We chose to use two-dimensional arrays for visualization in several places. These tables are highly suitable for comparisons of the properties of two drugs. Concepts are separated by changes in line thickness in the table. The table is thus divided into two parts, making it possible to differentiate between different types of data, such as quantitative data from clinical trials and the conclusions of experts.

We reduced the volume of text in the tables through the use of graphical representation in the form of pictograms.

We introduced modes of interaction with the user, providing physicians with easy access to more detailed sections from the global view. We ensured that the interface of the application did not appear overloaded, by displaying the value of cells through mouseovers (for example: for side effects, green indicates that the side effect was not reported).

### Identification of the corpus of information concerning the drug in the used sources

For data input into our system, we used the SPCs and evaluation reports of the French National Authority for Health (HAS). SPC information was extracted from the Claude Bernard database [15], which uses reference terminologies, such as MedDRA, ATC, IDC10, to index information contained in free text.

The characteristics of novelty of the drug were determined by considering all the drugs with the same indication described in the report of the French National Authority for Health (HAS) [13]. These drugs are presented by pharmacotherapeutic group in the report.

For each of these drugs, we used the SPC information available from the Claude Bernard database, including, in particular, the paragraphs concerning composition, pharmacodynamics, therapeutic indications, dosage, route of administration, technical and regulatory data (approval date, price, etc.).

We selected the comparator from the clinical trials described in the evaluation report of the French National Authority for Health (HAS). For a new drug for a given indication, in the best of cases, there is a comparator with the active ingredient; in most cases, the new drug is compared to placebo.

We used the results of clinical trials, based on the primary endpoints mentioned in the evaluation report [13], to describe the impact of the drug in terms of its efficacy. For description of the impact of the drug in terms of safety, we studied the following sections of the SPC of the new drug and its comparator: side effects, contraindications, warnings and precautions, drug interactions and overdose. The side effects are presented in MedDRA terms. The order of presentation of side effects was determined according to the System Organ Class (SOC) list of the MedDRA terminology, because this order is used internationally.

For the impact of the drug in terms of ease of use, we analyzed the paragraphs relating to dosage and mode of administration present in the SPC for the new drug and its comparator, as well as information from the evaluation report [13] concerning the conditions in which clinical trials were carried out.

We added some additional information to the items described in our previous study [12], to provide a more exhaustive view of the new drug. This new information included the date of marketing authorization, the daily cost of the drug and the target population.

For all items of the model relating to the impact of the new drug in terms of efficacy and safety, we listed all the possible values that the system could use for all new drugs.

### Methods used for system implementation

We developed the prototype in PHP and SQL. We selected five new pharmaceutical products with very different innovative characteristics, to illustrate the functioning of the prototype.

For each drug, we manually collected information from the SPC and evaluation reports, for the previously established elements. We created a database for the storage of all data on these products. The conclusions of the experts were extracted from the evaluation reports. The prototype uses the information provided by the French Claude Bernard drug database [15] and the evaluation report.

We applied several rules when generating the interface, to ensure homogeneity in terms of the representation of information. The routes of administration are represented by pictograms. The information about the new drug is always presented before the information about the drug with which it is compared. When the new drug is compared with placebo, for the safety and ease of use aspects, only information about the new drug is presented. The “serious drug-drug interactions” (SDDI) are presented in gray in the impact in terms of safety part of the interface if no SDDI has been reported.

### Method of evaluation

#### Overall approach

Our goal was to obtain a preliminary qualitative evaluation (except for the SUS score, which is semi-quantitative), to determine whether evaluators of various ages and medical backgrounds (i) appreciated this separation of drug information into three axes, (ii) had any suggestions for additional information to be displayed in the interface for each drug, (iii) appreciated the three levels of granularity of knowledge representation, (iv) found this prototype understandable and easy to use.

We defined several categories of physicians for this evaluation. We included 10 evaluators in this evaluation (two physicians highly specialized in the area most relevant to the drug (a Professor of Therapeutics and a Professor of Pharmacology at Paris 13 University), two university general practitioners (a Professor and an Assistant Professor in General Practice) particularly implicated in the field of therapeutics in primary care, two physicians (both Professors in Education Science carrying out research in the domain of therapeutic education), three young, less experienced GPs and a public health resident).

#### Choice of drugs

We chose five drugs recently approved in Europe and the USA and providing real innovations with respect to the comparator, either an active ingredient or placebo. These five drugs were identified by the French National Authority for Health (HAS) as being of “significant” actual benefit between 1 January 2008 and 1 April 2011. Efient® (prasugrel), Inovelon® (rufinamide), Gylenya® (fingolimod), Levact® (bendamustine) and Ellaone® (ulipristal), for which the improvement in actual benefit varied from “no improvement” to “modest improvement”. The therapeutic domain, type of treatment and type of novelty of the drug also differed.

#### Evaluation protocol

The evaluators were provided with a printed version of the evaluation reports for each of the five drugs. They also had a printed version of the SPCs of the drugs compared in the evaluation report. They had access to the software developed in this work.

The qualitative evaluation was carried out with a specific questionnaire (see Additional file 1).

The prototype was presented to the evaluators, who were then provided with an opportunity to use the prototype themselves. They were then asked to complete the questionnaire.

Physicians were also asked to complete the System Usability Scale (SUS) [16], a standard algorithm for evaluating the usability of web sites. For each question, a response is given on a scale of 1 to 5, according to the user’s degree of satisfaction.

## Results

### Graphical presentation

#### Overall approach

**Figure 1:**
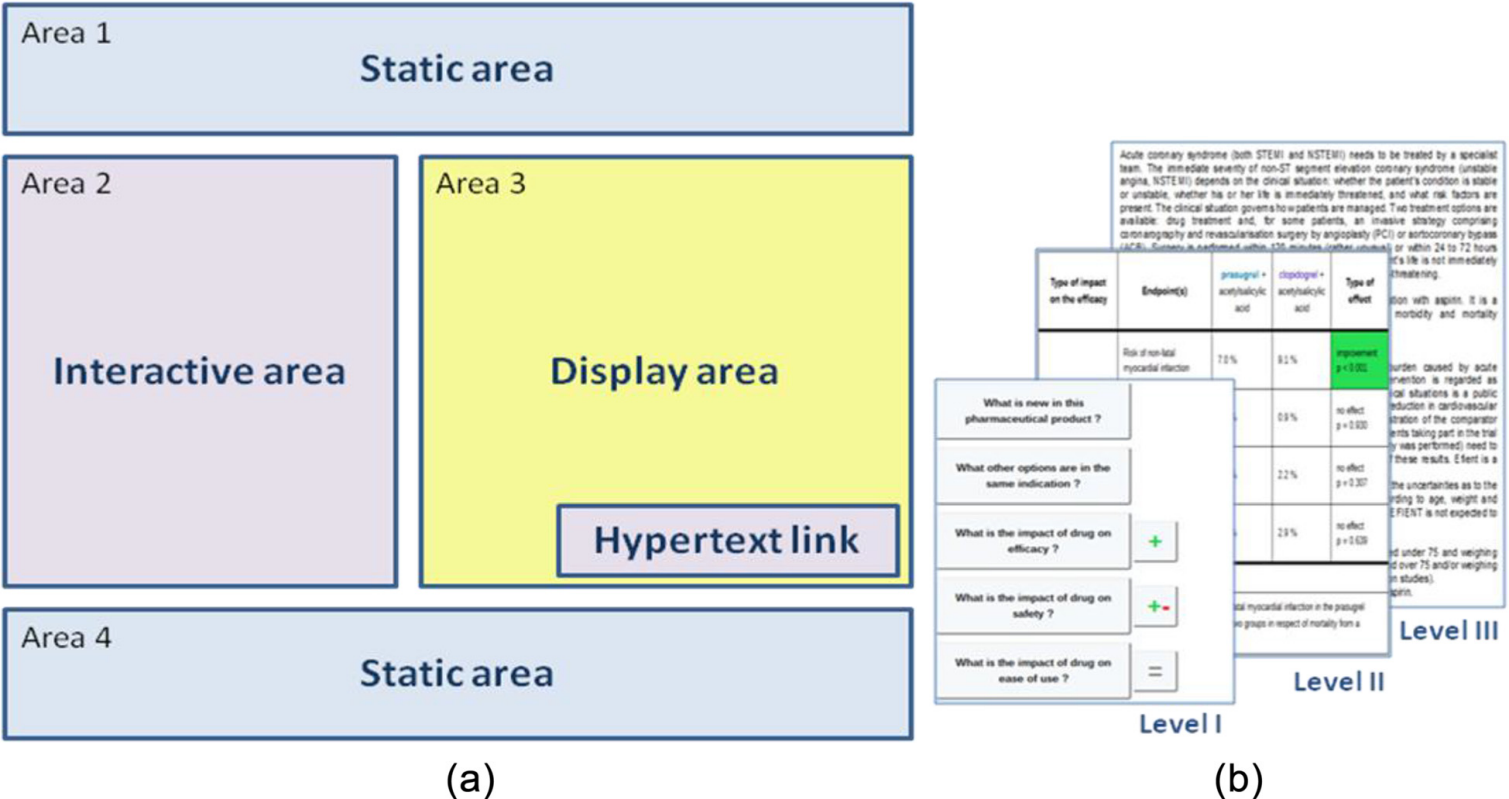
Illustration of the organization of information in the prototype. (**a**) – Area 1 includes the trade name of the drug, its dose and form, and its indication. Area 2 represents the axes of the conceptual model in the form of a menu containing highly synthetic information; Area 3 shows the synthetic information; Area 4 includes information about the target population, actual benefit, improvement in actual benefit, approval date and comparator. (**b**) – The three levels of information detail (I – highly synthetic, II – synthetic, III – detailed). Area 1, Area 4 and Area 2 corresponding to Level I from (**b**) are always present. The level II of granularity is shown in Area 3 when the physician chooses a topic from the menu. From level II, the user can obtain level III of detail by clicking on the hypertext link.

We chose to represent the display consistently, using the same stable elements and splitting the display into several areas, as shown in Figure 1. The elements of Figure 1B are displayed in Area 3 of Figure 1A. The static area at the top of the page contains information about the health problem, the exact indication for which the drug is prescribed. The static area at the bottom of the page contains information about the target population, the actual benefit (AB), the improvement of actual benefit (IAB), approval date and the comparator. These elements are present on each page. We ensured that the information presented in the figures remained readable, by extracting from the display only Area 3 for some figures. The interactive area is presented as a menu. Each element of the menu corresponds to an axis of the conceptual model described in detail elsewhere [12]. We chose to represent information according to three levels of detail: highly synthetic, synthetic and detailed. Clicking on one of the buttons in the display area reveals the second level of detail. The hypertext link area provides access to the most detailed level, corresponding to excerpts from the evaluation report [13] and SPC.

#### Description of the interface showing highly synthetic information

**Figure 2:**
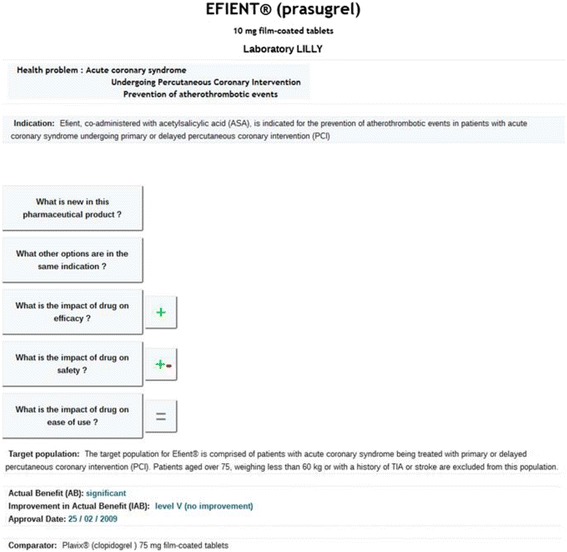
The highly synthetic level of the interface for Efient®.

Figure 2 shows an overview of the main features of the new drug Efient® (prasugrel), 10 mg, administered together with acetylsalicylic acid for the prevention of atherothrombotic events in patients with acute coronary syndrome undergoing primary or delayed percutaneous coronary intervention (PCI). This drug was authorized for market release on February 25, 2009. The actual benefit of Efient® was rated “significant.” However, this drug provided no improvement over the available therapeutic arsenal (level 5 of the French classification). Efient® (prasugrel, 10 mg) is compared to Plavix® (clopidogrel, 75 mg).

#### Description of the interface showing synthetic information

##### Novelty of the new drug

**Figure 3:**
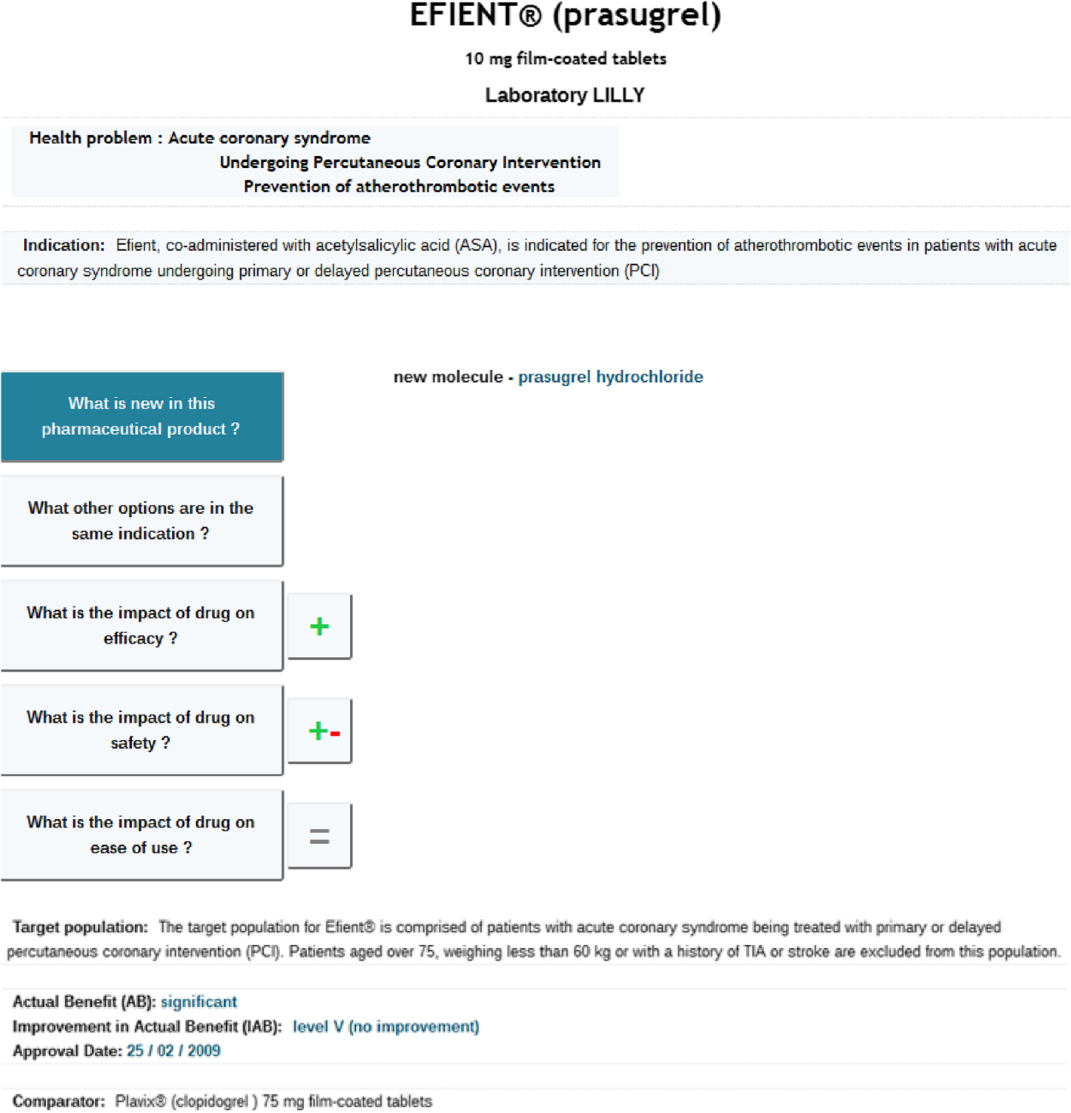
Representation of the novelty of Efient® (the synthetic level, Area 3). When the user clicks on the topic “What is new in this pharmaceutical product?” in Area 2, the feature(s) of novelty of this new drug is (are) displayed in Area 3.

The user must click on the corresponding item in the menu to find out what is new about the pharmaceutical product. For example, the novelty of Efient® lies in the presence of a new molecule, prasugrel hydrochloride. This is illustrated in Figure 3.

##### Other drug options with the same indication

The user must click on the corresponding item in the menu to find out what other options are available for the same indication.

We generated a table, in which products are grouped according to pharmacotherapeutic group and mechanism of action and the route of administration is shown, to provide a representation of all drugs with the same indication. Pictograms are used to represent the route of administration of the product to facilitate comparisons between drugs.

**Figure 4:**
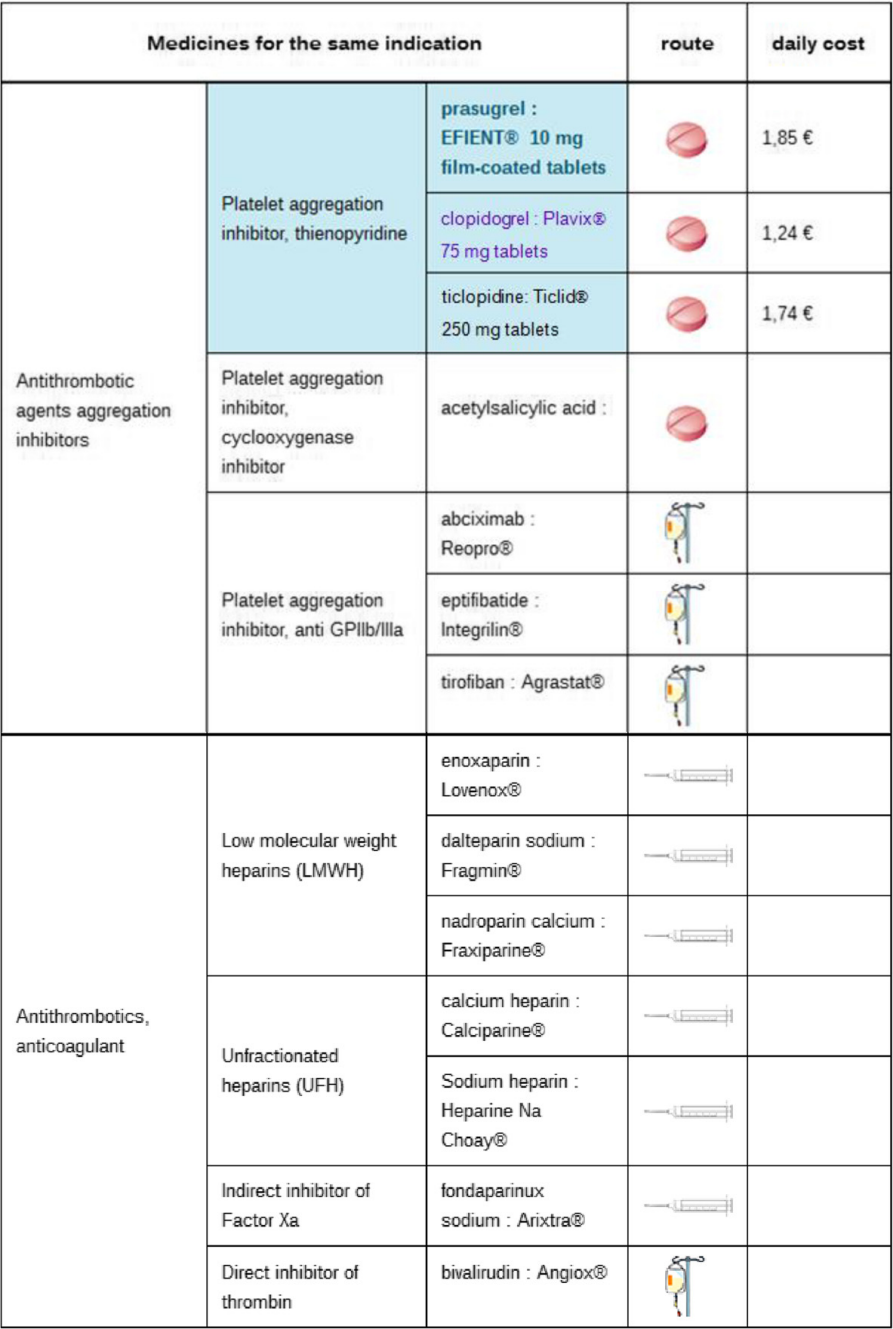
Screenshot showing all the drugs with the same indication as Efient® (prasugrel) (the synthetic level, Area 3). When the user clicks on the topic “What other options are in the same indication?” in Area 2, the table with all the drugs sharing the same indication is displayed in Area 3.

Figure 4 shows all the drugs prescribed for the same indication. Daily costs of the drug are presented if available. Unfortunately, this value cannot always be calculated, because the dose may depend on the weight or body surface area of the patient, for example.

##### Impact of the new drug in terms of efficacy

**Figure 5:**
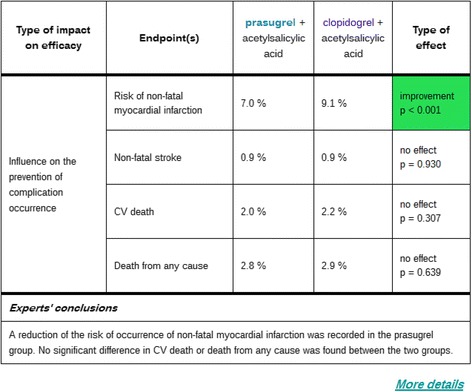
Screen shot showing the impact of Efient® on efficacy (the synthetic level, Area 3). When the user clicks on the topic “What is the impact of drug on efficacy?” in Area 2, a comparison of the efficacy of the two drugs is displayed in Area 3. The thick line separates the numerical values obtained in clinical trial from the experts’ conclusions coming from the evaluation report, which are shown at the bottom of the table.

We used tables to visualize the impact of the drug in terms of efficacy, safety and ease of use. For comparisons of the efficacy of the new drug and its comparator, the table includes the values of endpoints measured in clinical trials, the endpoints in a given trial being identical for the two drugs compared, and the probability value *p* quantifying the statistical significance of the type of effect. In cases in which this *p* value is less than 0.05, the existence of a significant difference is indicated in green. The thick line divides the numerical values obtained in clinical trials from the experts’ conclusions, which are provided at the bottom of the table and are taken from the evaluation report. The impact of Efient® in terms of efficacy is shown in Figure 5.

##### Impact of the new drug in terms of safety

**Figure 6:**
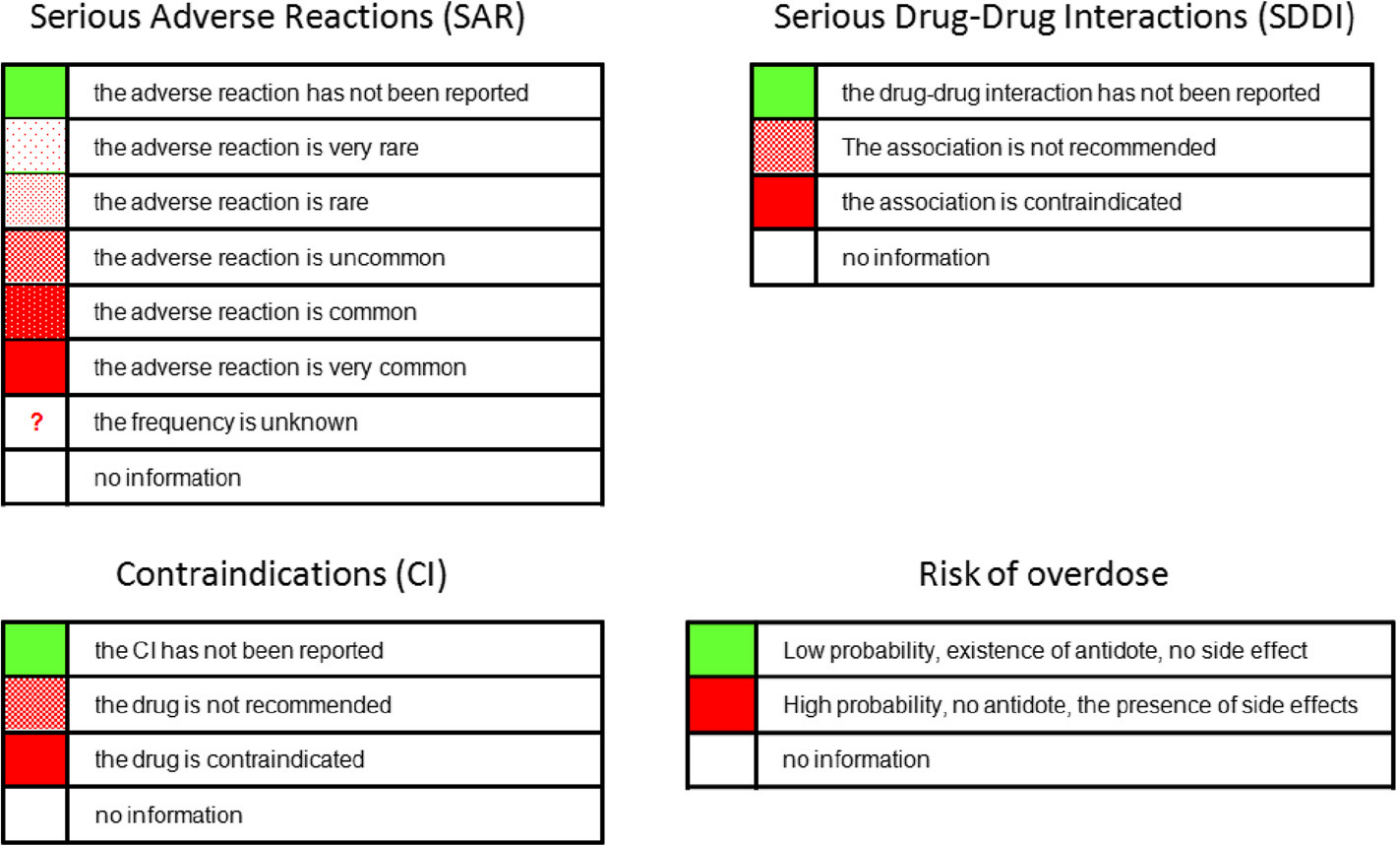
Ways of representing safety characteristics: serious adverse reactions, contraindications, serious drug-drug interactions, risk of overdose.

The use of the attribute “color” in the representation of the impact of the drug on safety makes it possible to represent various types of information within a single cell. Figure 6 shows the possible values for the frequency of serious adverse reactions, serious drug-drug interactions, contraindications and the risk of overdose.

**Figure 7:**
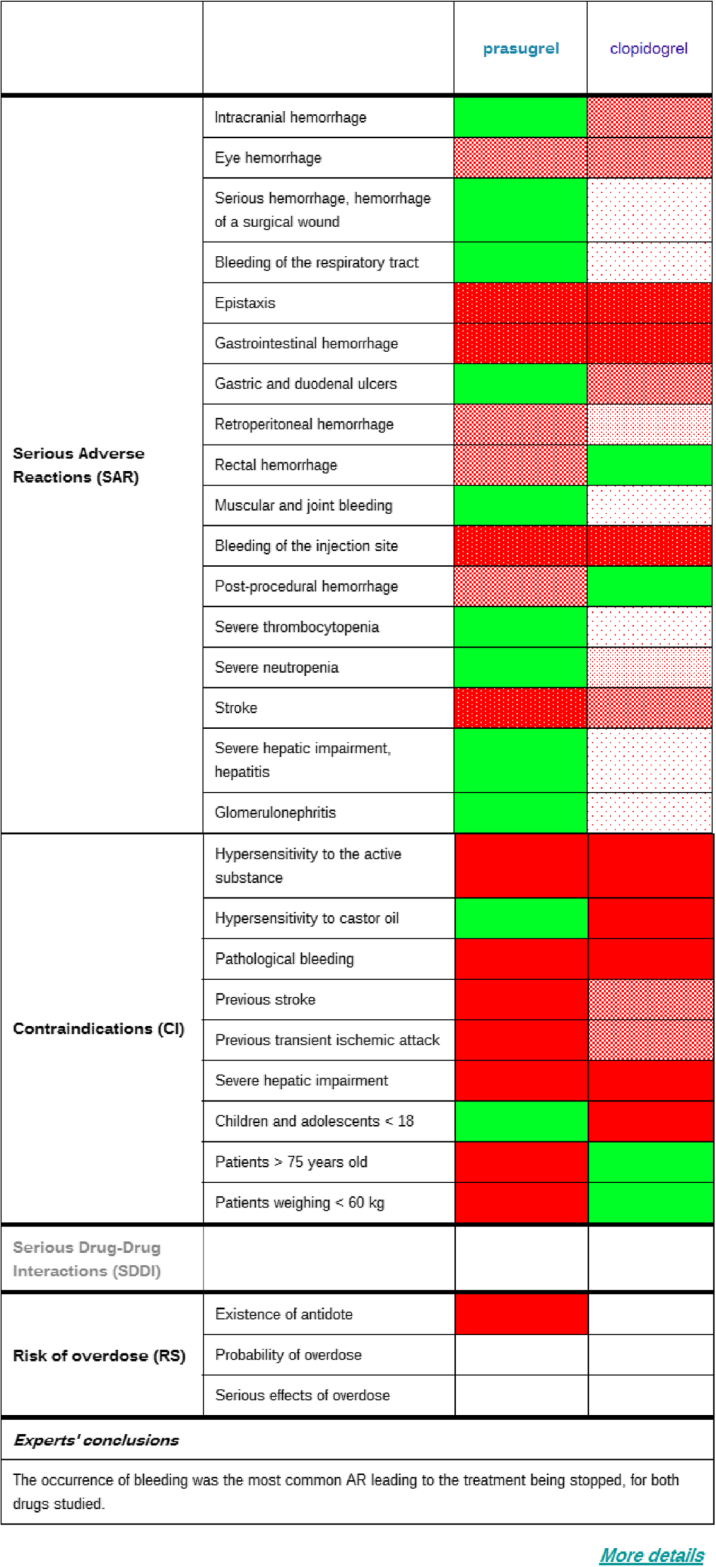
Screen shot showing the impact of Efient® on safety (the synthetic level, Area 3). When the user clicks on the topic “What is the impact of drug on safety?” in Area 2, a comparison of the safety of the two drugs is displayed in Area 3.

We took into account only “absolute” contraindications for the new drug and the comparator. Similarly, for drug-drug interactions, we considered only associations that are contraindicated. Figure 7 shows the impact of the drug Efient® on safety.

Serious adverse reactions, with frequency intervals from clinical trials, are shown at the top of the table. The conclusions of the experts, taken from the evaluation report, are shown at the bottom of the table, below the thick line.

##### Impact of the new drug in terms of ease of use

**Figure 8:**
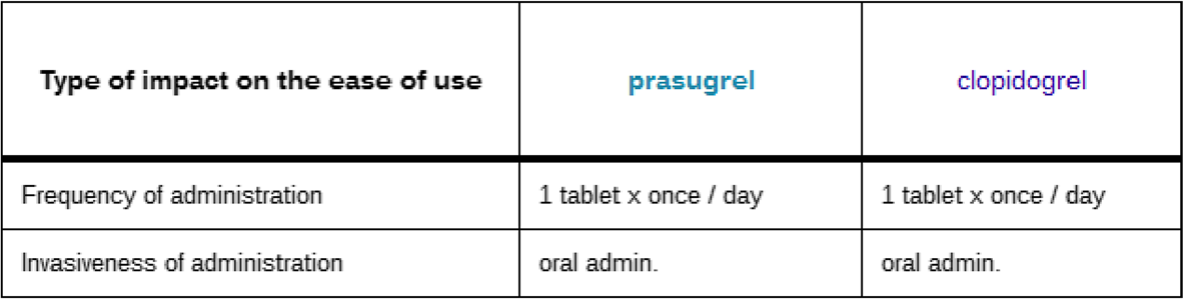
Screen shot showing the impact of Efient® on ease of use (the synthetic level, Area 3). When the user clicks on the topic “What is the impact of drug on ease of use?” in Area 2, a comparison of the ease of use of the two drugs is displayed in Area 3.

The information for each drug was derived from the description of the clinical study presented in the evaluation report [13] and the “Dosage and Administration” section of the SPC. We represented the impact of the new drug on ease of use, by considering the various items relating to this subaxis in our model. However, we display only information that is actually informative for the reader. For example, in Figure 8, for the new drug Efient*®,* we do not take into account items that are not relevant for the two drugs (e.g.: duration of administration for tablets, duration of an anticoagulant treatment, convenience of administration and adjustment of dose) or items not mentioned in the sources used (e.g.: complexity of treatment monitoring). There is no difference in ease of use between prasugrel and clopidogrel, both of which are taken once daily in tablet form.

### Description of the interface showing detailed information

#### Detailed information about efficacy

The doctor can access additional information about efficacy by clicking on “More details”. This brings up excerpts from the SPC and/or the evaluation report. When the user clicks on the hypertext link “More details” in Area 2, the detailed information about efficacy is displayed in a new window. For Efient®, the text issued from the efficacy subsection of the evaluation report of the Transparency Commission (22 July 2009) is the following:

*“The combination of prasugrel (EFIENT) + aspirin was associated with a significant reduction in ischemic events, including stent thrombosis, compared to the combination of clopidogrel (PLAVIX) + aspirin after a median follow-up of 15 months treatment. The difference in risk (absolute benefit) was 2.1% (9.4% versus 11.5% in the overall population, p < 0.001) in favour of the prasugrel group. This effect was essentially due to a reduction in the risk of experiencing a non-fatal myocardial infarction (9.1% in the clopidogrel group and 7.0% in the prasugrel group, p<0.001). No difference between the two groups was observed in mortality (from any cause or from a cardiovascular cause).”*

#### Detailed information about safety

As for efficacy, the physician can obtain additional information about safety, by clicking on the hypertext link “More details” in Area 2.

For Efient®, we give here three texts issued from the safety subsection of the evaluation report of the Transparency Commission (22 July 2009):

- *For serious adverse reactions*
  *“Premature cessation because of a bleeding event was more frequent in the prasugrel group (2.5%) than in the clopidogrel group (1.4%). A higher rate of bleeding was observed in the prasugrel group: major bleeding not related to CABG (2.17% in the prasugrel group and 1.65% in the clopidogrel group, p=0.029); potentially life-threatening bleedings (1.26% in the prasugrel group versus 0.83% in the clopidogrel group, 0=0.015) including fatal bleedings (0.31% versus 0.07%, p=0.002).*
  *In patients with a history of stroke or TIA the risk-benefit ratio of prasugrel (EFIENT) was unfavourable: treatment with EFIENT is contraindicated.”*

b) *For contraindications*
  *Both drugs are contraindicated in patients with pathological bleeding, as well as severe hepatic impairment.*
  *Contrary to clopidogrel, prasugrel is contraindicated in persons with known hypersensitivity to castor oil.*
  *For people with a history of transient ischemic attack and stroke, prasugrel is contraindicated, while clopidogrel is not recommended.*

c) *For Overdose*
  *“An overdose of prasugrel and clopidogrel may cause prolonged bleeding time and subsequent bleeding complications. There are no data on the neutralization or pharmacological effect of prasugrel or clopidogrel, but if a prompt correction of prolonged bleeding time is required, a platelet transfusion and/or other blood products may be considered.”*

### Evaluation of the prototype

All the physicians appreciated the representation of information according to three levels of granularity: highly synthetic, synthetic and detailed.

Eight physicians agreed with the choice of three axes for the description of pharmaceutical innovation (context, novelty and impact). A professor specializing in general practice suggested that more detail could be provided for the novelty axis, with a judgment on the results of completed trials, particularly when the novelty concerns a new molecule. A professor of education sciences suggested that the name “ease of use” was not ideal for the last axis, but he proposed no alternative formulation.

Six of the 10 physicians considered the two synthetic levels of granularity of the description sufficient for the rapid construction of an opinion concerning the drug. The others felt that all three levels of granularity were important for forming an opinion.

Nine of the 10 physicians found the comparison of the possible occurrence and frequency of serious adverse reactions easy to understand. The professor of general practice felt that it was important to specify the frequency of serious adverse reactions by giving the percentage recorded during clinical trials.

All doctors found the mode of comparison of contraindications and risks of overdose easy to understand. Eight physicians felt that the comparison of the efficacy of the new drug with that of its comparator was easy to understand. Eight physicians felt that it was easy to determine whether there were other drugs in the same therapeutic class for the same indication.

Some evaluators suggested adding information about the drug, such as the detailed dosage schedule and precautions for use (the professor of pharmacology and a professor of education science), the total cost of treatment (the professor of therapeutics), and the rate of reimbursement by the social security system (public health resident).

The time required to form an opinion about the potential value of a new drug was estimated at three minutes by three evaluators, at five minutes by two and at five to ten minutes by two evaluators.

One evaluator felt that it was impossible to get a clear idea from the sources used (SPC and evaluation report). He felt that there was a problem concerning the reliability of information. The mean score attributed by the 10 physicians was high, at 82, corresponding to a “good” user interface.

## Discussion

On the basis of these findings, we propose the selection, grouping and presentation of types of knowledge relating to pharmaceutical innovations, to provide physicians with easily accessible, objective information about new drugs. The design of the prototype presented here was based on modeling of the main elements of pharmaceutical innovations identified in our recent study. Our objective was to provide physicians (mostly GPs) with a tool enabling them to decide rapidly whether a new drug is of potential interest for use in their practices.

We have taken into account the limitations to the amount of information a user can examine and process at a given moment due to his or her cognitive and perceptual abilities [17]. Physicians thus require an overall idea of the utility of the new drug, based on an overview of its main properties. We therefore propose three levels of granularity, from highly synthetic to detailed.

It has been shown [18] that standardized and consistent interfaces are required for users to be able to master their use without special training. For this reason, we chose to represent the interface with stable elements, to avoid the need for learning.

We used graphical representation in the form of pictograms, because it has been shown that users prefer graphical interfaces, which facilitate learning to a greater extent than purely textual interfaces [19].

We considered only the texts available from the national drug agencies at the time of market release. These texts are restricted to the evaluation reports written by the experts working for these agencies and the texts of the SPC for the new drug and the drugs with which it is compared. We did not take scientific articles reporting the results of the trials conducted for evaluation of the new drug into account, because we considered the reading of these texts to be too time-consuming for physicians.

At the most detailed level, our prototype provides excerpts of the evaluation reports written for the French National Authority for Health (HAS). We could also have considered evaluation reports prepared in other countries, which would be particularly interesting in cases in which opinions differ. We plan to tackle this aspect in future studies.

We used the results of clinical trials to describe the impact of the drug in terms of efficacy. The comparator was identified from clinical trials. For comparisons of the safety of the new drug with its comparator, we restricted the analysis to serious adverse events, serious drug-drug interactions, contraindications and the risk of overdose. This made it possible to focus on the most important aspects of the pharmaceutical innovation, rather than providing doctors with too much information, which might deter them from reading. As we had mentioned in our previous work [12], we compare the new drug to existing drugs with exactly the same indication. The therapeutic arsenal is identified for this indication in evaluation reports. If the drug is approved for many indications, a separate representation is made for each indication.

We show only the serious adverse reactions defined precisely in the study [20] on the basis of explicit criteria. This choice facilitates the selection of adverse reactions to be included in the system for each new drug.

Our visual approach, combining color and texture, seems particularly appropriate for the comparison of side effects, which may occur with both new and old drugs, because a side effect may be present or absent and, if present, its frequency may vary. We did not take color blindness (an inability to distinguish between red and green) into account. However, when the user places the mouse over the colored area, a text providing a description is displayed.

Our approach closely resembles that of Duke *et al*. [21], who proposed data visualization methods for the representation of potential adverse drug events in patients on multiple medications.

Our interface was designed to compare only two drugs at a time, but this is not a major limitation, because (i) most clinical trials compare no more than two drugs, (ii) for safety evaluations, our prototype could easily be adapted for the comparison of more than two drugs, by adding additional columns if similar types of data are available for all the drugs.

Our method of presenting information draws attention to the drugs that are the first to be released for a given indication. If there are no other drugs for the indication concerned, then the drug may be considered truly innovative. In such cases, the information is less rich for safety and ease-of-use representation, because there is no comparator. Similarly, if the comparator is a placebo, there are no comparative data concerning safety and ease of use.

The evaluation was carried out by GPs and highly qualified professionals specializing in particular areas, with different skills, to provide us with a diverse range of opinions concerning the various elements and the classification of items. We now plan to ask GPs to carry out a more specific evaluation, in which we will compare the time required for a GP to answer questions about a new drug correctly with the full-text documents and with the software.

The information visualization procedure provided by this prototype software was much appreciated by the physicians participating in its evaluation. None of these physicians felt that any internationally important information was missing and should be added, but the addition of nation-specific information should be considered if this prototype is to be adapted for use in other countries. The decision to present information at multiple levels of detail was also particularly appreciated by the evaluators, who found the prototype easy to understand and to use.

More detailed information could have been provided on the first screen. However, we chose to provide only the information permitting the physician to determine very quickly whether the new drug was likely to be of any value for his or her own practice.

It is also easy to add new drugs to this system. Information can be introduced into the system by a pharmacist without programming skills. For each drug, it takes between a half day and two days to add the information required, depending on the number of drugs in the therapeutic arsenal and the number of adverse reactions to the new drug and its comparator.

## Conclusion

Overall, we believe that this work can serve as a template for efforts to provide objective information about new drugs from health agencies, training organizations, medical journals and the pharmaceutical industry, which might be interested in rationalizing the information provided to potential prescribers of new products.

## Additional file

Additional file 1:
**Questionnaire for the qualitative evaluation.**
