# Supplementary material for: Design and evaluation of a software for the objective and easy-to-read presentation of new drug properties to physicians
Source: BMC Med Inform Decis Mak. 2015 May 30;15:42. doi: 10.1186/s12911-015-0158-2 (PMC4460682; doi:10.1186/s12911-015-0158-2)
Supplement: Additional file 1: — Questionnaire for the qualitative evaluation. [file 12911_2015_158_MOESM1_ESM.doc]

1. Do you appreciate the representation of information about each new drug in three levels of granularity (from the most synthetic to the most detailed)? Yes No

*Comments and suggestions…………………………………………………………………………*

1. Do you agree with the choice of three axes of description of pharmaceutical innovation (context, novelty and impact)? Yes No

*Comments and suggestions…………………………………………………………………………*

1. Do you agree with the choice of three sub-axes of description of the impact (efficacy, safety and ease of use)? Yes No

*Comments and suggestions…………………………………………………………………………*

1. Can you propose other types of information to be added to each drug?

*Suggestions…………………………………………………………………………………………..*

1. Do the first two levels of granularity of the description already allow you to quickly form an opinion on the study drug? Yes No

*Comments and suggestions…………………………………………………………………………*

1. Do you understand the comparison of the possible occurrence and frequency of serious adverse reactions? Yes No

*Comments and suggestions…………………………………………………………………………*

1. Do you understand the modalities of comparison of contraindications? Yes No

*Comments and suggestions…………………………………………………………………………*

1. Do you understand the comparison of the risks of overdose? Yes No

*Comments and suggestions…………………………………………………………………………*

1. Do you understand the comparison of the efficacy of the new drug and its comparator?

Yes No

*Comments and suggestions…………………………………………………………………………*

1. Is it easy with this presentation to know if there are other drugs in the same therapeutic class in the same indication? Yes No

*Comments and suggestions…………………………………………………………………………*

1. Would you have preferred that the set of the information presented on the complete interface be shown in gray? Yes No

*Comments and suggestions…………………………………………………………………………*

1. Do you have any suggestions to improve the interface? Yes No

*Suggestions*…………………………………………………………………………………

1. How much do you estimate the time you need to form an opinion on the interest of a new drug with this tool?
